# Human Immunodeficiency Virus (HIV) Disclosure and Sexual Behaviors among Kenyan Women

**DOI:** 10.16966/2380-5536.165

**Published:** 2019-05-16

**Authors:** Luz A Padilla, Tierra Johnson, Rubi Galarza, Haley Greene, Gerald Mc Gwin, Pauline E Jolly

**Affiliations:** Department of Epidemiology, School of Public Health, University of Alabama at Birmingham, Alabama, USA

**Keywords:** HIV/AIDS, HIV disclosure, Women, Sexual risk behaviors, Kenya

## Abstract

**Background:**

Disclosure of HIV status is crucial to the adoption of preventive behaviors for HIV transmission. This study was conducted to investigate HIV status disclosure and its impact on sexual practices among HIV-positive women in Nairobi, Kenya.

**Methods:**

A cross-sectional study was conducted among HIV-positive women seeking care at two hospitals in Nairobi. A questionnaire and known self-efficacy scales were administered to eligible women to collect information on sociodemographic factors, HIV disclosure and beliefs, healthcare provider advice on disclosure, sexual practices, and self-efficacy.

**Results:**

Of 497 women who were included in this analysis, 95.8% had disclosed their HIV status to someone. Women who disclosed were more likely to be in a relationship (p 0.0017) and to be the heads of their households (p 0.0042). Women who reported that their healthcare provider advised them to disclose and told them of ways to disclose were significantly more likely to have disclosed (p=0.0237 and p=0.0294, respectively). The belief that HIV status disclosure is important for HIV prevention and control and that the benefits of disclosure outweigh the risks was also significantly associated with disclosure (p<0.0001 for both).

**Conclusion:**

The prevalence of disclosure among HIV-positive individuals’ in hospital settings in Nairobi is high. These findings indicate that encouraging and suggesting ways to disclose by healthcare providers and individuals’ beliefs about the benefits of disclosure can increase the rate of HIV disclosure. Prospective studies to assess these observations would provide reliable guidance on how to increase disclosure by all women.

## Introduction

Human Immunodeficiency Virus (HIV) status disclosure is a sensitive and complicated process due to social, financial, and personal consequences that can result; yet, disclosure is critical to the prevention of HIV and care of those infected [1,2]. HIV disclosure is important as it has been associated with a reduced risk of HIV transmission, especially to sexual partners of infected individuals, and may lead to the adoption of preventive behaviors for the transmission of sexually transmitted diseases (STDs) [1,2]. Studies on HIV status disclosure in Africa indicate that it is highly variable depending on the source and recipient of the disclosed information; rates of disclosure to sexual partners is particularly highly variable (10–80%) [1,3–9]. In Africa, the rates of HIV disclosure by women to their sexual partners are especially lower when compared to HIV disclosure by men to their sexual partners; this is partly due to socio-cultural and financial implications including fear of discrimination, anticipated disruption of relationships or even abandonment, fear of emotional and physical abuse, and wanting to avoid being stigmatized [1,2,8–11].

Kenya has the fourth largest HIV epidemic in the world, with an overall HIV prevalence of 4.8% and an estimated 28,000 deaths from AIDS-related sickness in 2017 [12,13]. The HIV epidemic in Kenya is thought to affect all sections of society including children, young people, and adults [12]. However, recent reports have indicated that Kenyan women are among the most vulnerable groups affected by HIV [14]. It is estimated that about 29% of female sex workers and 7% of the overall female population in Kenya have HIV [14,15]. HIV disclosure among women may actually impact the rates of HIV infection among the general population, yet it is not commonly done in many areas of sub-Saharan Africa [16–18]. The purpose of this study is to determine the prevalence of HIV disclosure among HIV-positive women in Kenya and, identify specific characteristics among HIV-positive women who have not disclosed. The findings may help in designing new, cost effective strategies and improve already existing public health programs in Kenya to further increase rates of disclosure.

## Materials and Methods

A cross-sectional study was conducted among 502 HIV-positive women seeking out-patient care at Kenyatta National Hospital (KNH) and Mbagathi District Hospital (MDH) in Nairobi, Kenya in 2012. KNH is the largest referral hospital in the country with an 1800 bed capacity and serves as the primary hospital for the 4 million residents in Nairobi. MDH is a smaller hospital with only 200 beds that provides health services to 9800 HIV-positive individuals and is the tuberculosis referral center for Nairobi as well. The study participants were HIV-positive women within the reproductive age-range (19–49 years), who were sexually active (had sex in the past 6 months), not currently pregnant, had a CD4 count of ≥ 350 cells/cm^3^ blood, and were free of any AIDS defining illness (HIV stage I and II). In addition, these women had to be willing to consent to the abstraction of data from their medical records. Institutional Review Board approval was obtained prior to the implementation of this study.

HIV-positive women who met the inclusion criteria were identified by the clinic staff who informed them of the study and asked if they would be willing to participate. Women who were willing to participate were then introduced to the study team. Following informed consent, an interviewer-administered questionnaire was used to collect information on sociodemographic factors, disclosure of HIV-positive status, beliefs regarding the risk/benefit of disclosure, advice by a Healthcare Provider (HCP) to disclose, abuse by partner, and sexual behaviors. The questionnaire also contained known scales that are related to HIV disclosure; namely, Kalichman’s HIV status disclosure and safer sex practice negotiation ability self-efficacy scale, the Multidimensional Scale of Perceived Social Support (MSPSS), and The Thailand stigma scale [19–21].

The medical records of the participants were reviewed to obtain clinical information including HIV diagnosis date, viral load if available, and CD4+ count.

### Kalichman: HIV status disclosure self-efficacy scale and safer sex self-efficacy scale

The Kalichman scales are valid and reliable tools used to assess one’s efficacy on disclosing HIV status to sexual partners and ability to negotiate openness to safer sex practices among seropositive people. It can be used to predict, describe, and improve self-efficacy for the behaviors measured [19].

### Multidimensional Scale of Perceived Social Support (MSPSS)

The MSPSS is used to assess differences in social support that is perceived from family, friends, and partners by an individual. This scale helps in understanding these relationships, as social support can be essential in improving one’s mental and physical health. The MSPSS has been shown to have excellent consistency test-retest validity and internal reliability (Cronbach alpha 0.81–0.98 in non-clinical samples, and 0.92–0.94 in clinical samples) [20,21].

### Thailand stigma scale

The Thailand stigma scale measures stigma of HIV and TB not only in Thailand but in developing countries at the community and individual levels with the intention of creating new interventions that will decrease the burden of stigma and shame related to these diseases. This scale proved to be internally consistent, valid, showed test-retest reliability, and includes standardized summary scores. It has been re-validated in among non-Hispanic Whites, non-Hispanic Blacks, Hispanics, and non-Hispanic other, living with HIV in the United States [22,23]. However, we believe it has not been validated in African populations.

### Statistical analysis

Sociodemographic, health and sexual behavior characteristics were compared according to HIV disclosure status. HIV disclosure status was defined by participants’ self-reported answer to the question, “Have you disclosed your HIV status to anyone?” Differences between those who responded “Yes” and “No” were compared using chisquare, Fisher’s exact, and t-tests, where appropriate. Variables with significant p-values < 0.05 were used to estimate crude odds ratios and 95% confidence intervals of the predictors or associated variables for disclosing HIV status using logistic regression. An adjusted model was not possible due to the small numbers in our HIV non-disclosure group. SAS 9.4 (SAS institute, NC) software was used for the analysis and all statistical tests of a two-sided p-value of <0.05 were considered significant.

## Results

A total of 502 participants were recruited, of which 497 completely filled the questionnaire and had accessible medical records. Of the 497 women, 476 (95.8%) reported that they had disclosed their HIV status to someone while 21 (4.2%) had not. Among those who disclosed their HIV status, 47.1% were married or lived with a partner and 52.9% reported being single, divorced or widowed; however, all women in this study had been sexually active within the last 6 months. Approximately 34% of the women reported that they had disclosed their HIV status to their main sexual partner, 54% had disclosed to a religious leader, 7% to a family member other than their spouse/sexual partner, and 2% had disclosed to a friend. Most of the 21 women who had not disclosed (85.7%) said they did not have a partner and the other 14.3% said they were in a relationship (p 0.0017). Fifty-eight percent of participants who had disclosed their HIV status reported that they were the heads of their households compared to 95% of those who had not disclosed (p 0.0042). Of those who had not disclosed, 5% had a parent as the head of the household, compared to 7.6% of those who had disclosed. Of those who had disclosed, 24.1% reported a partner and 10.3% reported other as heads of their households (other includes other family members such as uncles, aunts, grandparents, and friends). No other sociodemographic variables were significantly associated with HIV disclosure status in this study (Table 1).

Table 2 shows participant’s CD4 counts, beliefs about the risk/benefit of disclosure, sexual behavior variables, and report of advice by an HCP to disclose, by disclosure status. Among those who had disclosed their status, the majority (90.9%) reported that their HCP advised them to disclose compared to 57.1% among those who had not disclosed (p=0.0237). Furthermore, 90.1% of those who had disclosed their HIV status reported that their HCP explained ways in which they could disclose compared to 57.1% of those who had not disclosed (p 0.0294). Participants who disclosed their HIV status believed that disclosing is important for HIV prevention and control (90.1%), whereas only 56.3% of those who had not disclosed believed this to be true (p<0.0001). Most participants who had not disclosed their HIV status reported that they did not think that the benefits outweighed the risks of disclosure (71.4%), compared to 20.1% of those who had disclosed (p<0.0001). Seventy-three percent of participants who had disclosed reported knowing their partner’s HIV status compared to 26.7% of those who had not (p 0.0003).

When the Kalichman scale, the MSPSS scale, and the Thailand stigma scale were used to evaluate HIV disclosure by the women, significant differences were obtained for the Kalichman HIV status disclosure self-efficacy scale and the MSPSS scale (Table 3). The mean total score for the Kalichman HIV disclosure self-efficacy scale among the women who had disclosed was twice as much (14.97 ± 5.2) when compared to women who had not disclosed (6.43 ± 2.7) (p<0.0001). The mean score for the MSPSS scale was 52.95 ± 17.4 for those who had disclosed their HIV status compared to 33.1 ± 18.6 for those who had not disclosed (p<0.0001).

The crude odds ratios and 95% confidence intervals (CI) for significant variables in the bivariate analyses are presented in table 4. Women who were advised and told of ways to disclose their HIV status by their HCP were close to 7 times more likely to disclose (OR7.53, CI1.58–35.85 and OR6.84, CI1.45–32.41, respectively). Beliefs that disclosure was important and that the benefits of disclosure were greater than the risks significantly increased odds of disclosure (OR15.41, CI1.58–35.85 and OR6.84, CI1.45–32.41, respectively).

## Discussion

Our study, conducted at two high level urban hospitals in Kenya, showed that HCPs might possibly influence an individual’s HIV status disclosure. These particular hospital settings may impact disclosure rate and cause it to be over reported, thus disclosure reported in this study may not reflect the rate of the larger population or the rate in smaller clinics and rural settings. However, high specialty hospitals also have a higher patient load, which may decrease the time spent by HCPs with every individual discussing the importance of disclosure and information related to their HIV status. Previous studies in Africa targeted behavior changes such as regular condom use and decreasing stigma as means to increase disclosure [24,25]. The findings of this study suggest that in addition to existing efforts, programs that help HCPs become aware of how important it is to encourage individuals to disclose while also providing possible ways in which to do so would be beneficial. These programs should stress the importance of disclosing for HIV prevention and control in order to change an individual’s misperceptions on the benefits of disclosure [26].

The results from this study suggest that HCPs may be associated with an individual’s probability of disclosing their HIV status. The findings in table 4 show that the odds of disclosing are over 6 times higher among those who were told to disclose and of ways to disclose. Also, as shown in table 2, the proportion of being advised by a HCP to disclose was significantly higher among those who had disclosed their HIV-positive status when compared to those who had not (90.9% *vs* 57.1%, respectively); it is important to add that in the overall cohort less than half (45%, 225/497) were advised to disclose. Making HCPs aware of this as well as ensuring their proper training to converse and educate HIV-positive individuals about disclosure may help to increase disclosure among these individuals. HCPs may also be a good source to discuss the risks and benefits of disclosure with HIV-positive individuals. However, other sources of communication and new public health programs that educate and decrease stigma and fears while highlighting the benefits of disclosure may also modify these beliefs. Increased implementation of disclosure self-efficacy scales to people after their HIV-positive diagnosis may be useful in providing insight on fears and beliefs that may impede individuals from disclosing. This may also allow HCPs’ and public health programs to be more specific in targeting the barriers to disclosure based on low scores on certain items on these scales by particular participants and could make interventions more cost-effective.

The majority of participants who had disclosed were married or in a common-law relationship. This may influence disclosure since the partner may have been the source of infection and may be more apparent in a common living situation or stable relationship. Participants who knew their partner’s HIV status were over 7 times more likely to disclose their HIV status. However, this may be because participants who had not disclosed were not in a stable or committed relationship, as all participants in this study reported being sexually active. This is consistent with findings from the 2012 South African National HIV Prevalence, Incidence and Behaviour survey, where the analysis showed that those who were not married, including those living in common-law relationships, were 60% less likely to disclose their HIV-positive status when compared to those who were married [27,28].

Participants who disclosed their HIV-positive status were 15 times more likely to report that HIV disclosure is important for HIV prevention and control, and 10 times more likely to state that the benefits of disclosure outweigh the risks. Participants who had not disclosed their status to anyone felt that disclosing their status was not beneficial; it is possible that these were individuals who were more likely not to be in a committed relationship. This was observed in a previous study, which found that family members and partners provided the most moral support after disclosure [29]. Furthermore, individuals who are not in a committed relationship may also be the same ones that do not believe that disclosing will help HIV prevention and control and, may also believe that disclosing does not outweigh the risks. Support for this is seen in the significantly lower scores from the MSPSS among those who have not disclosed their HIV-positive status as this scale is used to grade the participants’ perception of social support (i.e., from family and friends).

The score for the self-efficacy Kalichman scale which assesses the confidence and ability to disclose by participants was twice as high among those who disclosed, further analysis showed that for a one unit increase in a participant’s score, the chance of disclosure increased by 30%. Additionally, our assessment of the belief of the importance and benefits of disclosure was 9 and 15 times higher among those who had already disclosed. The combination of participants not knowing the benefits and importance of sharing their HIV status with their partners, and not being confident or able to disclose, is particularly concerning. Studies have shown that independently building confidence and self-esteem as well as increasing knowledge of how to live successfully with HIV, may not only increase disclosure but may also have a synergistic effect in helping the individual with decreasing stigma, increasing life satisfaction, and quality of life [30,31]. Unreported analysis on self-efficacy scores showed that 30–40% of those who did not believe that disclosure of HIV status was important or beneficial either strongly disagreed or disagreed with being confident or able to disclose (p<0.0001). Interventions that help individuals who show poor confidence and acknowledge their inability to disclose while being unaware of the benefits of disclosure should be established. These programs can be implemented or led by HCPs and include family and friends. Additionally, using known scales that evaluate disclosure may further increase the chances of disclosing. Scales maybe used as tools to possibly identify low scores on specific items by individuals who are less likely to disclose, perhaps this may lead to more targeted, meaningful, and cost-effective interventions.

A limitation of this study is that the population was drawn from hospitals, which may not reflect disclosure from people who seek care in smaller institutions, rural areas, or people who do not seek care at all; these hospital participants may have overestimated the prevalence of disclosure when compared to the general public. People with HIV who attend a hospital may feel more comfortable with their diagnosis or may be more ill, either situation may influence their decision to disclose. People who seek healthcare or attend their doctor’s appointments are the people who have already disclosed. Future studies should implement strategies that recruit individuals outside of hospitals in both rural and urban settings. A major limitation of our study analysis is that due to the small numbers in the non-disclosure group an adjusted model was not possible, this may affect the true strength of associations and conclusions reported and limits accounting for other confounding factors. Another major limitation is the cross-sectional nature of our study design that does not allow us to interpret our results as cause and effect and only shows disclosure prevalence at one point in time. Our small sample size in the non-disclosure group affected comparability between the two groups. Additionally, this study may have social desirability bias where subjects may have over-reported disclosure in order to be viewed favorably; disclosure status was obtained through self-reporting which is also open to bias and was not confirmed.

## Conclusions

Disclosure of HIV status has been shown to prevent and help to control the transmission of HIV. The prevalence of disclosure among HIV-positive individuals in high-level hospital settings in Kenya is high but may not reflect the disclosure status among the general population. This study suggests that HCPs are a crucial group influencing HIV disclosure status. Additionally, changing patient’s misperceptions on the benefits and importance of disclosure and increasing confidence in their ability to disclose may be some elements that may further increase the chances of disclosure. Known scales for evaluating disclosure may possibly be used as tools to identify those individuals with low scores on specific items related to attitudes, knowledge, or self-confidence as those who are less likely to disclose; this may lead to individualized, meaningful, and cost-effective interventions that target such characteristics. However, further studies that sample populations from urban and rural areas and in and out of hospital settings are needed in order to make reliable conclusions on what is deterring some individuals from disclosing their HIV-positive status.

## Figures and Tables

**Table 1:** Sociodemographic variables by HIV disclosure.

Variables	Yes N (%)	No N (%)	p-value
**Age**			0.256
19–30	78 (16.4)	1 (4.8)	
31–40	251 (52.7)	12 (57.1)	
≥41	147 (30.9)	8 (38.1)	
**Marital status**			**0.002**
Married/Common Law	222 (47.1)	3 (14.3)	
Single/Divorced/Widow	249 (52.9)	18 (85.7)	
**Number of persons in household**			0.593
<5	221 (46.4)	11 (52.4)	
≥5	255 (53.6)	10 (47.6)	
**Social status**			0.577
High	9 (1.9)	0 (0)	
Average	305 (64.3)	15 (71.4)	
Low/Below poverty line	160 (33.8)	6 (28.6)	
**Education**			0.214
None/Primary	125 (26.3)	8 (38.1)	
Secondary	220 (46.2)	9 (42.9)	
Tertiary	131 (27.5)	4 (19.1)	
**Employment status**			0.589
Employed/Self-employed	335 (70.8)	16 (76.2)	
Unemployed	138 (29.2)	5 (23.8)	
Religion			1
Christian	460 (97.5)	21 (100)	
Other/Muslim/None	12 (2.5)	0 (0)	
**Head of household**			**0.004**
Self	275 (58.0)	19 (95.0)	
Sexual partner/spouse	114 (24.1)	0 (0)	
Father/Mother	36 (7.6)	1 (5.0)	
Other	49 (10.3)	0 (0)	
**How much older is your partner/spouse than you**			0.424
<10	195 (42.0)	10 (47.6)	
≥10	94 (20.3)	2 (9.5)	
Same age	175 (37.7)	9 (42.9)	
**Residential area**			0.991
Rural	71 (15.3)	3 (14.3)	
Urban	392 (84.3)	18 (85.7)	

* Numbers may not add to total number of participants due to missing responses; Bold=significant at p<0.05

**Table 2:** Participants’ mean CD4 counts, beliefs about the risk/benefit of disclosure, sexual behavior, and report of advice by healthcare provider to disclose, by HIV status disclosure.

Variables	Yes N (%)	No N (%)	p-value
**Age**			0.256
19–30	78 (16.4)	1 (4.8)	
31–40	251 (52.7)	12 (57.1)	
≥41	147 (30.9)	8 (38.1)	
**Marital status**			**0.002**
Married/Common Law	222 (47.1)	3 (14.3)	
Single/Divorced/Widow	249 (52.9)	18 (85.7)	
**Number of persons in household**			0.593
<5	221 (46.4)	11 (52.4)	
≥5	255 (53.6)	10 (47.6)	
**Social status**			0.577
High	9 (1.9)	0 (0)	
Average	305 (64.3)	15 (71.4)	
Low/Below poverty line	160 (33.8)	6 (28.6)	
**Education**			0.214
None/Primary	125 (26.3)	8 (38.1)	
Secondary	220 (46.2)	9 (42.9)	
Tertiary	131 (27.5)	4 (19.1)	
**Employment status**			0.589
Employed/Self-employed	335 (70.8)	16 (76.2)	
Unemployed	138 (29.2)	5 (23.8)	
Religion			1
Christian	460 (97.5)	21 (100)	
Other/Muslim/None	12 (2.5)	0 (0)	
**Head of household**			**0.004**
Self	275 (58.0)	19 (95.0)	
Sexual partner/spouse	114 (24.1)	0 (0)	
Father/Mother	36 (7.6)	1 (5.0)	
Other	49 (10.3)	0 (0)	
**How much older is your partner/spouse than you**			0.424
<10	195 (42.0)	10 (47.6)	
≥10	94 (20.3)	2 (9.5)	
Same age	175 (37.7)	9 (42.9)	
**Residential area**			0.991
Rural	71 (15.3)	3 (14.3)	
Urban	392 (84.3)	18 (85.7)	

* Numbers may not add to total number of participants due to missing responses; Bold=significant at p<0.05

**Table 3:** HIV disclosure scale scores by HIV disclosure.

Scales	Yes	No	p-value
	**Mean** ± **SD**	**Mean** ± **SD**	
**Kalichman Scales**			
HIV status disclosure self-efficacy scale			
Total Score	14.97 ± 5.2	6.43 ± 2.7	**< 0.0001**
Safer sex self-efficacy scale			
Total Score	18.19 ± 3.7	16.69 ± 4.5	0.165
**Multidimensional scale of perceived social support parameter**			
Total score	52.95 ± 17.4	33.10 ± 18.6	**<0.0001**
**Thailand stigma scale**			
Perceived stigma			
Total Score	11.53 ± 6	9.1 ± 2.5	0.064
Internalized shame			
Total Score	15.71 ± 8.8	16.14 ± 8.6	0.828

* Numbers may not add to total number of participants due to missing responses; Bold=significant at p<0.05

**Table 4:** Odds of disclosing HIV status to main sexual partners.

Variables	Crude OR (95% CI)
**A health care provider told me to disclose my HIV Status**	
Yes	**7.53 (1.58–35.85)**
No	Ref
**A health care provider explained ways that I could disclose my HIV status**	
Yes	**6.84 (1.45–32.41)**
No	Ref
**Do you believe HIV disclosure is important for HIV prevention and control**	
Yes	**15.41 (5.25–45.24)**
No	Ref
**Do you think the benefit of HIV disclosure outweigh the risk**	
Yes	**9.92 (3.04–32.34)**
No	Ref
**Do you know the HIV status of your sexual partner**	
Yes	**7.51 (2.34–24.15)**
No	Ref
**Kalichman Scale**	
HIV Status disclosure self-efficacy scale	*1.36 (1.11–1.68)
Multidimensional scale of perceived social support parameter	*1.08 (1.05–1.12)

* Continuous variables
